# Perspective on 3D vertically-integrated photonic neural networks based on VCSEL arrays

**DOI:** 10.1515/nanoph-2022-0437

**Published:** 2023-01-13

**Authors:** Min Gu, Yibo Dong, Haoyi Yu, Haitao Luan, Qiming Zhang

**Affiliations:** Institute of Photonic Chips, University of Shanghai for Science and Technology, Shanghai 200093 China; Centre for Artificial-Intelligence Nanophotonics, School of Optical-Electrical and Computer Engineering, University of Shanghai for Science and Technology, Shanghai 200093 China

**Keywords:** diffractive neural networks (DNNs), integrated photonic neural network, three-dimensional (3D) photonic chip, vertical-cavity surface-emitting laser (VCSEL)

## Abstract

The rapid development of artificial intelligence has stimulated the interest in the novel designs of photonic neural networks. As three-dimensional (3D) neural networks, the diffractive neural networks (DNNs) relying on the diffractive phenomena of light, has demonstrated their superb performance in the direct parallel processing of two-dimensional (2D) optical data at the speed of light. Despite the outstanding achievements, DNNs utilize centimeter-scale devices to generate the input data passively, making the miniaturization and on-chip integration of DNNs a challenging task. Here, we provide our perspective on utilizing addressable vertical-cavity surface-emitting laser (VCSEL) arrays as a promising data input device and integrated platform to achieve compact, active DNNs for next-generation on-chip vertical-stacked photonic neural networks. Based on the VCSEL array, micron-scale 3D photonic chip with a modulation bandwidth at tens of GHz can be available. The possible future directions and challenges of the 3D photonic chip are analyzed.

## 1 Introduction

With the rapid development of artificial intelligence (AI), traditional complementary metal oxide semiconductor (CMOS) electronic chips based on von Neumann architectures gradually could not satisfy the computational power required by AI training tasks. According to a report by OpenAI [1], after 2012, the computational power required to train the largest AI program will double every 3.4 months. Due to the gradual failure of Moore’s law [2], and the serial nature of von Neumann architectures where the memory and processor are separated [3], current AI training and operational tasks require large numbers of GPUs (graphics processing units) and CPUs (central processing units), lead to an astronomical amount of time and energy costs [4]. Therefore, developing non-von Neumann computing systems are of great importance for various AI applications, such as autonomous vehicles, facial recognition, and robotics.

Photonic neural networks (PNNs), which mimic the key functions of biological neuronal systems, are regarded as a promising next-generation computing architecture [3, 5]. Compared with electrons, photons have more degrees of freedom (wavelength, phase, polarization and angular momentum), enabling high-bandwidth data transmission. As a data carrier, photons travel at the speed of light with extremely low delay which is 300 times faster than that of electrons [6]. Since the propagation of light is passive, the calculation is completed after the light propagates through the predefined structures [5]. Therefore, PNNs have extremely low energy consumption which is mainly concentrated in the laser source, the synaptic weight modulation, and the output signal reading. Theoretically, the potential processing speed of PNNs can reach petaMAC/s/mm^2^ (MAC: multiply-accumulate operations) with an energy efficiency of attojoule/MAC [2, 7]. On the contrary, the maximum processing speed of von Neumann electronics is only at the level of GMAC/s/mm^2^ with picojoule/MAC energy efficiency [2].

Integrated on-chip and compact sized PNNs are becoming an attractive field. The emergence of two-dimensional (2D) PNNs based on Si-photonics, where the light propagates within a plane, have demonstrated their versatility in numerous AI applications, including image classification [8], vowel recognition [9] and unsupervised correlation detection [10]. Through the utilization of Mach–Zehnder interferometers [9], phase change materials with photonic circuits [11, 12], and micro-ring resonators [13], various integrated PNNs have been achieved including deep neural networks [9, 14], spiking neural networks [15], tensor cores [16] and convolutional accelerators [17], showing the advantages of neuromorphic photonic computing. However, the 2D cascade architecture of PNNs based on Si-photonics set a natural constraint on their intelligence and parallelism. As PNNs based on Si-photonics have a limited wafer size [9, 15], and the size of photonic components cannot be much smaller than the working wavelength [18], making it impossible to realize large number of synaptic connections on a Si-wafer. Additionally, the cascade design of photonic circuits may lead to uncontrollable noises/errors in the PNNs when scaling up [9].

Recently, three-dimensional (3D) PNNs based on the diffraction phenomenon of light have proved to be a promising solution: diffractive neural networks (DNNs). The concept of 3D PNNs based on diffractive holograms was proposed in the 1990s [19, 20], and firstly realized in the terahertz range in 2018 [21]. The DNN is physically created by using several cascaded transmissive and/or reflective layers, where the incoming wave propagates in free space, and each pixel on a given layer either transmits or reflects the incoming wave, representing an artificial neuron that is connected to other neurons of the following layers through optical diffraction. Due to the 3D architecture, DNNs have demonstrated their superb performance in direct parallel processing of two-dimensional data without the need to convert the data to sequential inputs as in 2D PNNs [16]. Important applications, such as image classification [21–25], optical logic operation [26, 27], image reconstruction [28] and terahertz pulse shaping [29], have been successfully demonstrated with DNNs. Given 3D two-photon nanolithography (TPN) is applied, 500 million neurons/cm^2^ can be easily achieved in D^2^NNs [22, 30].

However, current DNNs may not suitable for on-chip integration applications. This is because current DNNs usually take advantage of centimeter-scale devices to generate the input data passively, such as, spatial light modulators (SLM), digital-micromirror devices (DMD), or diffractive plates [21–26], which results in the spatial separation of signal generator and DNNs, and causing the size of entire optical setup at centimeter level or even meter level. Therefore, developing novel DNNs architecture with active inputs at micrometer scale becomes an inevitable pathway for next generation on-chip PNNs.

## 2 VCSEL array for 3D photonic neural networks

A vertical-cavity surface-emitting laser (VCSEL) is a kind of semiconductor laser first invented in 1970s [31]. The structure of a typical VCSEL from top to bottom is usually P-type distributed Bragg reflectors (P-DBR), active layer and N-DBR grown on a III-V semiconductor substrate [32]. As a semiconductor laser source, a VCSEL has the advantages of small size (μm-scale), low threshold, controllable high modulation bandwidth (more than 30 GHz) [32–34], and has been widely used in field of optical communication, optical storage and sensing [32]. More importantly, the perpendicular emitting phenomenon of VCSEL makes it convenient to realize a laser array [35–37], which can be a perfect candidate as active input source for DNNs. The addressability of VCSEL arrays enables the realization of optical inputs (images) for DNNs, where each VCSEL in the array can be independently controlled through independent electrodes [37].

**Figure 1:**
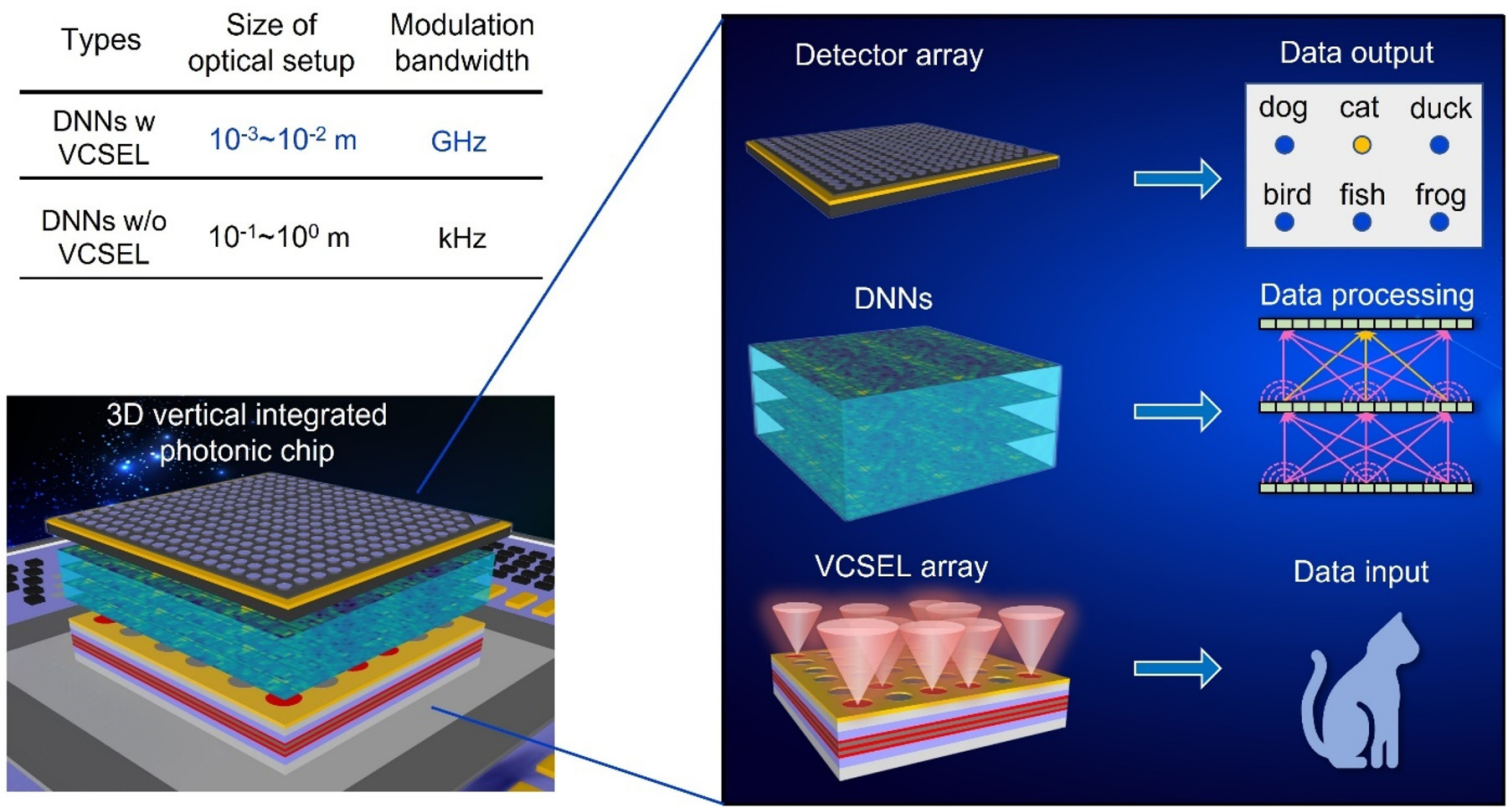
The on-chip integration design of DNNs based on VCSEL arrays.

Therefore, it is necessary to initiate an exciting journey to utilize addressable VCSEL arrays as a promising integrated platform to achieve compact, active DNNs for next-generation on-chip vertical-stacked PNNs. The introduction of addressable VCSEL arrays can replace both the centimeter-scale solid/gas lasers and signal generator (DMD) in the existing DNNs optical setup to generate two-dimensional optical data for DNNs. As shown in Figure 1, based on the advanced bonding techniques, all devices for DNNs can be vertical stacked and on-chip integrated, and a sandwich structure of VCSEL array, DNNs and detector arrays can be constructed to fully realize a 3D photonic chip. In this perspective design, an array of VCSELs functions as the input ports in the DNN. The designed DNNs perform corresponding tasks for different input signals. The detector array can be integrated on the DNNs to receive the output data of DNNs.

Addressable VCSEL array pitches are typically hundreds of microns [36, 37]. When used for image recognition, take 28 × 28 pixels handwritten digit images in MNIST (Modified National Institute of Standards and Technology) database as an example [38], 28 × 28 scale VCSEL array can meet the needs of optical image data input. Therefore, the size of this chip can be as small as several millimeters. The addressable driving of a small-scale VCSEL array can be achieved through existing electrical chips, like field programmable gate array (FPGA) chip, but when the scale of VCSEL arrays increases, designing dedicated driver chip will be essential [36]. We can learn the electrical control from micro-light-emitting diodes array [39]. The number of connections between a neuron in the previous layer and neurons in the next layer is a key parameter determining the performance of DNNs. To achieve as many as connections, reducing the neuron size by using TPN or bonding the diffractive layers with a designed substrate thickness together may be possible solutions. Of course, the implementation of VCSEL based DNNs presents unprecedented science and engineering challenges afar current technologies. For example, the superposition of coherent light is the basis in current DNNs, so the realization of coherence between the VCSELs in an array is important. To achieve this, anti-waveguide may be a feasible way [40, 41]. On the other hand, although the optical signals between each VCSELs are generally incoherent, the signal emitted by each individual VCSEL is coherent, therefore, there is another possible way to realize VCSEL-based DNNs by developing new design algorithms of DNNs.

## 3 Discussion and future perspectives

The future directions of VCSEL-based 3D photonic chips could be more intelligent, more compact, and higher speed (Table 1). More intelligent features means that DNNs can have higher accuracy, handle more complex tasks and more types of tasks. Programmability allows the chip to complete more types of tasks. In this concept, the VCSEL array can not only be used to generate signals, but also to regulate the synaptic weights of DNNs. To achieve this, novel photonic memristive materials [12] for DNNs should be encompassed. The programmable layer can be placed at the forefront of DNNs to facilitate VCSEL control. Meanwhile, multi-task learning algorithms of DNNs also need to be constructed. Multiplexing can increase the bandwidth of DNNs, which is one of the main advantages of PNNs. To enable the VCSEL array to generate optical signals with more dimensions, such as phase and orbital angular momentum, the coupled VCSEL array [41] and the *meta*-VCSELs [42] may be considered. The VCSELs can also be used as spiking neurons [43, 44] to further develop spiking DNNs, which is much closer to the operation form of human brain. In addition, micro- and nano-lasers may also be considered as spiking neurons to form spiking DNNs [45]. Compared with VCSELs, micro- and nano-lasers have smaller size, which can achieve higher neuron density.

**Table 1:** Future directions of VCSEL-based 3D photonic chip.

Future directions	Potential features needed	Key requirements	Applications
More intelligent	Programmable	Photonic memristive materials (phase change materials, photochromic materials, etc.)	Optical computing (matrix multiplication, logic operation)
				Image classification
	Multiplexing (phase, OAM, etc.)	New designs of a VCSEL array	Face recognition	
					Automatic driving
	Spiking DNNs	New algorithms			
			6G communication
		New integration architecture		Optical encryption
	Nonlinear activation function	Nonlinear optical materials		
	Higher neuron density	Advanced lithography technology, advanced micro–nano fabrication technology
More compact	CMOS-chip-controlled addressable VCSEL array	Back-emitting VCSELs
	Long-term stability	Heat dissipation
Higher speed	Higher operating speed	High-speed VCSELs
		High- speed photodetectors

At present, there is a lack of nonlinear activation function in the operation of DNNs, which limits DNNs to become deep DNNs. Therefore, the insertion of one or more nonlinear optical layers between the diffractive layers is important. A higher neuron density, which requires the introduction of more advanced lithography and fabrication technology, can enable DNNs to perform more complex tasks. The DNNs on the VCSEL arrays can be made by 3D printing or semiconductor manufacturing on transparent substrates, such as quartz or sapphire [46].

For being more compact, a flip-chip bonded VCSEL array can be the best choice, in which the back-emitting VCSEL arrays can be bonded onto a CMOS chip to solve the problem of the complex addressing circuits for a large-scale VCSEL array [36]. Another advantage is that the back-emitting VCSEL used in flip-chip bonding has a flat emitting surface, which is benefit for continue vertical integration of DNNs [42]. As the scale of VCSEL arrays increases, the heat dissipation of the VCSELs will be a concern. Excessive temperature can cause changes in the laser power and wavelength, thus affecting the accuracy of DNNs. To solve this, in addition to using conventional heat sinks, the introduction of microfluidics [47] or films with high thermal conductivity, like graphene [48], can also be considered to help the heat dissipation of the VCSELs. Besides, because DNNs can be integrated on the VCSEL array surface, some materials with high thermal conductivity can be chosen to fabricate DNNs.

For higher speed, since DNNs are passive, the operating speed is limited only by the signal input rate of the VCSELs and the readout rate of the photodetectors. Therefore, the further progress on the high-speed VCSELs and photodetectors is essential.

It can be envisaged that the integration scheme of DNNs and VCSEL arrays can be an advanced route of 3D photonic chips with a high feasibility and a wide application prospect. In the future, this chip can be applied to important fields such as optical computing (matrix multiplication and logic operation), image classification, face recognition, automatic driving, 6G communication, and optical encryption. With full play to the high modulation bandwidth of the VCSEL arrays, the task execution speed of this chip can be several orders of the state-of-the-art electronic chips with obvious advantages in parallel operation and energy consumption.
